# Successful Derivation of Hepatoblasts, Cholangiocytes and Hepatocytes from Simian Induced Pluripotent Stem Cells

**DOI:** 10.3390/ijms231810861

**Published:** 2022-09-17

**Authors:** Eleanor Luce, Clara Steichen, Soumeya Abed, Anne Weber, Philippe Leboulch, Leila Maouche-Chrétien, Anne Dubart-Kupperschmitt

**Affiliations:** 1 Unité Mixte de Recherche (UMR_S) 1193, INSERM/Université Paris-Saclay, F-94800 Villejuif, France; 2 Centre Hépatobiliaire, Fédération Hospitalo-Universitaire Hépatinov, Hôpital Paul Brousse, F-94800 Villejuif, France; 3 Division of Innovative Therapies, Institute of Biology François Jacob, INSERM, Paris-Saclay University, CEA Fontenay aux Roses, F-92260 Fontenay-aux-Roses, France; 4 Genetics Division, Brigham & Women’s Hospital and Harvard Medical School, Boston, MA 02115, USA; 5 Laboratory of Molecular Mechanisms of Hematologic Disorders and Therapeutic Implications, INSERM UMR 1163, Imagine Institute, Paris-Centre University, F-75015 Paris, France

**Keywords:** simian induced pluripotent stem cells, hepatocyte differentiation, cholangiocyte differentiation

## Abstract

The use of primary cells in human liver therapy is limited by a lack of cells. Induced pluripotent stem cells (iPSCs) represent an alternative to primary cells as they are infinitely expandable and can be differentiated into different liver cell types. The aim of our work was to demonstrate that simian iPSCs (siPSCs) could be used as a new source of liver cells to be used as a large animal model for preclinical studies. We first differentiated siPSCs into a homogenous population of hepatoblasts (siHBs). We then separately differentiated them into hepatocytes (siHeps) and cholangiocytes (siChols) expressing respective specific markers and displaying epithelial polarity. Moreover, we showed that polarized siChols can self-organize into 3D structures. These results should facilitate the deciphering of liver development and open the way to exploring co-culture systems that could be assessed during preclinical studies, including in autologous monkey donors, for regenerative medicine purposes.

## 1. Introduction

Many liver diseases can progress to acute liver failure, a critical situation where the only curative treatment is orthotopic liver transplantation (OLT). Because the number of available grafts remains insufficient, hepatocyte transplantation has been developed as an alternative to OLT [1]. However, the availability of human liver tissue, the difficulties encountered in isolating viable cells, their inability to proliferate in vitro and their difficult cryopreservation limit the therapeutic use of primary liver cells [2].

We therefore need to design new research models to improve our understanding of the biology of normal and pathological livers; this would enable the development of more accurate prognoses and more effective therapies [3]. However, our current knowledge of liver diseases is limited by genetic and environmental factors and the heterogeneous nature of each disease in terms of pathogenesis and response to therapy. Finally, the lack of well-characterized animal models for a large number of liver diseases remains a major obstacle.

Most of these animal models were developed in mice or rats, for obvious management and handling reasons but the significant genetic and phenotypic differences between rodents and humans are often responsible for clinical trial failures. Comparative genomic studies have shown that the genomes of humans and several non-human primates (NHPs) are highly conserved throughout evolution [4]. However, only very few NHP liver cell lines, such as immortalized fetal liver stem cells [5,6], are available for in vitro studies. Furthermore, because these cells have been modified to proliferate indefinitely and display a different gene expression profile compared to their in vivo counterparts, they cannot be used for clinical applications.

As for human cells, the directed differentiation of simian pluripotent stem cells offers a valuable alternative to these liver cell lines. Directed differentiation protocols were then used to obtain liver cells derived from human and simian embryonic stem cells (ESCs) [7]. Although there are gaps in the literature regarding the ethics of using animal stem cells for human benefit, research studies on these cells still raise questions regarding the ethics of harvesting stem cells from non-human beings [8].

The revolution triggered by the development of a protocol to reprogram somatic cells into induced pluripotent stem cells (iPSCs) [9], avoiding the specific ethical problems associated with the use of ESCs, was rapidly applied to monkey cells. The first simian iPSCs (siPSCs) were reported in 2008 [10]. Since then, many siPSC lines have been established [11,12]. It was shown that siPSCs proliferate and differentiate in a similar way to human iPSCs, which thus enabled the adaptation of many human cell protocols for monkey cells [13]. However, to our knowledge, the directed differentiation of siPSCs into hepatic cells has not yet been reported. This study describes the first successful approach to the generation of functional hepatocytes (siHeps) and cholangiocytes (siChols) from siPSCs.

## 2. Results

### 2.1. Differentiation of siPSCs into siHBs

**Figure 1:**
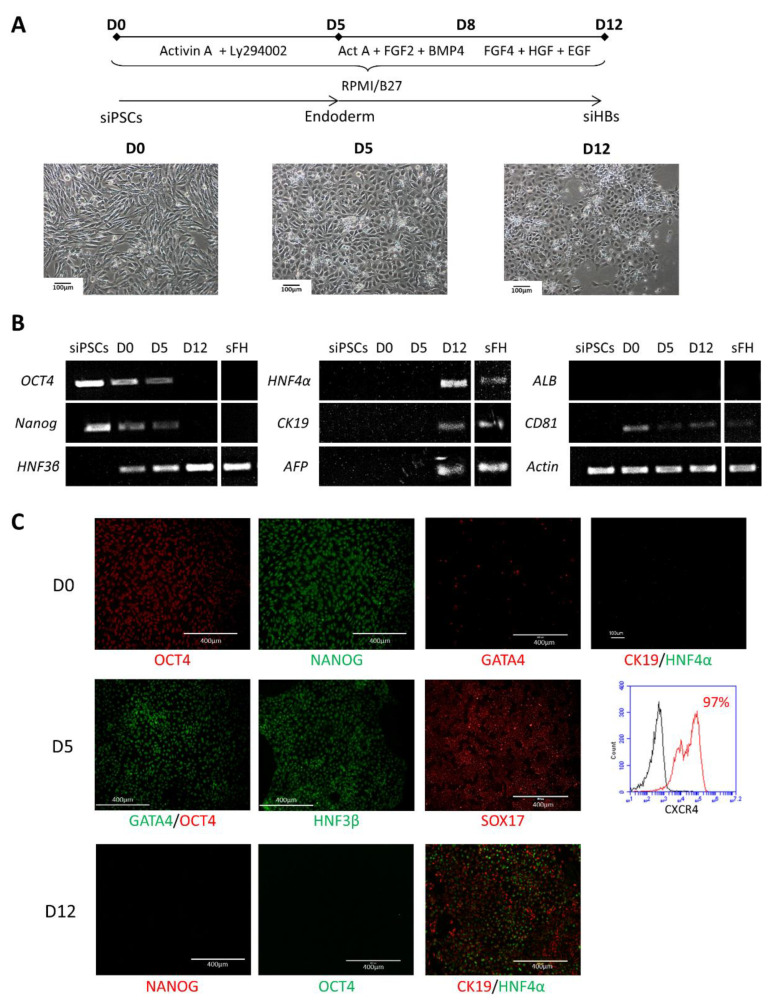
Hepatoblast differentiation of siPSCs. (**A**) Schematic representation of the protocol and phase contrast images illustrating the evolution of cell morphology. (**B**) RT-PCR showing modifications to the gene expression pattern during siPSC differentiation into siHBs. (**C**) Immunostaining and flow cytometry analyses showing the expression of OCT4 and NANOG stem cell markers, the definitive endoderm markers GATA4, HNF3β and CXCR4, and HNF4α and CK19 hepatoblast markers during siPSC differentiation. sFH: simian fetal hepatocytes.

We optimized previously published protocols developed on human cells [14,15] in order to differentiate siPSCs into siHBs (Figure 1A). siPSCs were cultured with Activin A and the inhibitor of phosphatidylinositol 3 kinase, Ly294002. This led to the formation of definitive endoderm cells expressing HNF3β, GATA4 and CXCR4 (Figure 1B,C) (see Tables S1 and S2 for the lists of antibodies and primers used). The cells were then cultured for three days with FGF2 and BMP4 to specify the definitive endoderm. After four additional days in the presence of FGF4 and HGF, the bipotent hepatic progenitors, siHBs, expressed HNF3β and HNF4α (hepatic nuclear factor 3β and 4α), AFP (alpha-fetoprotein), and CK19 (cytokeratin 19) (Figure 1B,C). Furthermore, the cells no longer expressed the pluripotency and stem cell markers OCT4 and NANOG (Figure 1B,C).

### 2.2. Differentiation of siPSCs into siHeps

**Figure 2:**
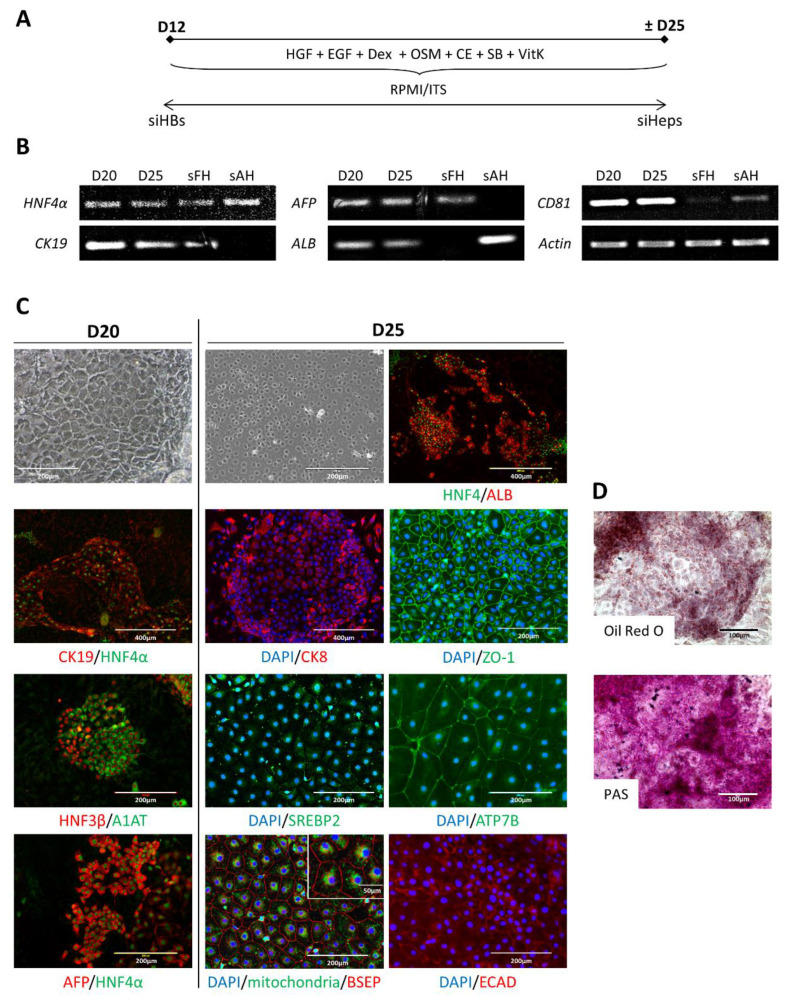
siHB differentiation into siHeps. (**A**) Schematic representation of the differentiation protocol. (**B**) RT-PCR showing the gene expression pattern during siHB differentiation into siHeps. (**C**) Immunostaining showing the expression of hepato-specific markers in siHeps. (**D**) Oil Red O and Periodic Acid Schiff (PAS) stainings of siHeps, evidencing lipid and glycogen storage, respectively. sAH: simian adult hepatocytes; sFH: simian fetal hepatocytes.

Cells were passaged on collagen-I-coated plates and treated for two days with DEX (dexamethasone), HGF, OSM (oncostatin M), TGFβ and CHIR99021 to promote their proliferation. The cells were then treated with inhibitors of the Notch and TGFβ pathways, both involved in cholangiocyte lineage specification, using Compound E and SB431542, respectively. We also treated the cells with vitamin K1 because 1% of the total proteins synthesized by the liver are vitamin K-dependent [16] (Figure 2A). Gene expression analysis performed on days 20 and 25 indicated the expression of the hepatic markers CK19, AFP, HNF4α, albumin (ALB) and CD81, an entry factor for Hepatitis C virus (Figure 2B).

From days 20–25, the morphology of siHeps closely resembled that of primary hepatocytes. Most cells expressed the hepato-specific markers HNF4α, AFP, CK19, HNF3β, A1AT, ALB, CK8, E-cadherin, the copper transporting ATPase ATP7B and the key cholesterol-metabolizing transcription factor SREBP2 (Figure 2C). siHeps displayed signs of polarity, as shown by the expression of the tight junction protein ZO-1 and of the bile salt export pump (BSEP), an efflux transporter that plays an important role in removing bile salts from hepatocytes into the bile canaliculi (Figure 2C). Furthermore, the siHeps harbored numerous mitochondria, a feature of hepatocytes that enables critical metabolic functions. Some of the hepatocyte functions have also been visualized such as the lipid and glycogen storage using PAS and Oil Red O staining, respectively (Figure 2D). Moreover, for future cell transplantation applications in animal models, the differentiated cells can be genetically marked by GFP-expressing lentivectors for better in vivo post-transplant follow-up (Supplementary Materials Figure S3).

Because the applications of iPSC-derived cells increasingly involve the use of 3D structures, and as we and others have shown the improved hepatocyte differentiation of human iPSCs in 3D organoids compared to 2D monolayer [17], we investigated the ability of siHeps to form spheroids. siHBs were seeded in inert agarose wells and then cultured using the same differentiation protocol as described for adherent cells (Supplementary Materials Figure S2A). The cells aggregated in the microwells and then clumped together to form a dark ‘core’ (Supplementary Materials Figure S2B). The cells composing the aggregates expressed hepatocyte markers such as ALB, HNF4α and HNF3β and seem to acquire a complex polarization highlighted by the expression of BSEP (Supplementary Materials Figure S2C). In addition, the cells harbored numerous mitochondria and highly expressed markers of mature hepatocytes, such as one of the major cytochromes P450 (CYP3A4) and CK8.

### 2.3. Differentiation of siPSCs into siChols

**Figure 3:**
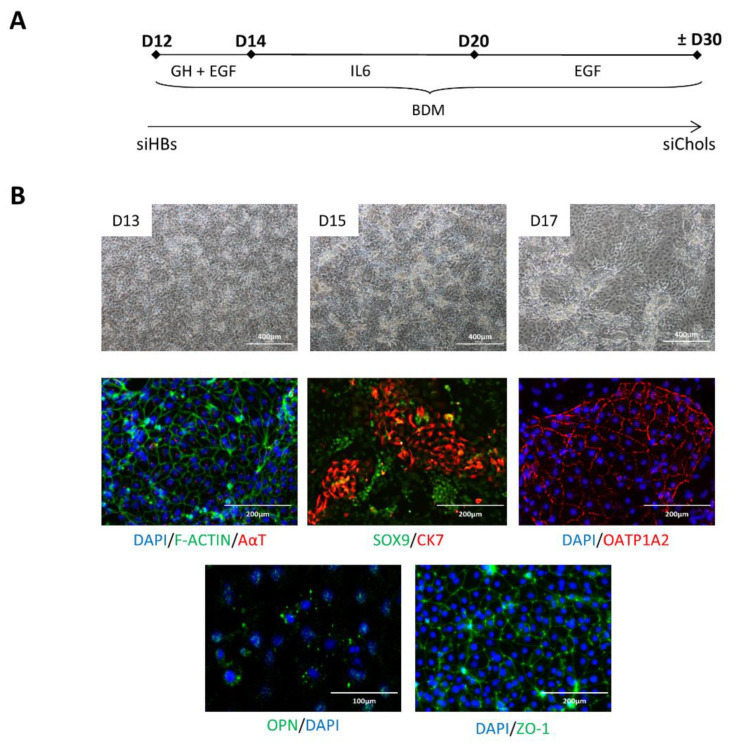
siHB differentiation into siChols. (**A**) Schematic representation of the differentiation protocol. (**B**) Cell morphology and immunostaining showing the expression of cholangiocyte-specific markers in siChols. BDM: biliary differentiation medium.

siHBs were treated for two days with EGF and growth hormone (GH), a regulator of the insulin-like growth factor-1 pathway. The cells were then incubated with interleukin-6 (IL6) (present in the fetal liver and involved in regulating biliary epithelial growth) and then supplemented with EGF until the end of differentiation (Figure 3A). At day 17, the cells expressed SOX9 (a transcription factor required for bile duct development) and osteopontin (OPN). Furthermore, the siChols also expressed OATP1A2, the organic anion transporting polypeptide 1A2, expressed in the apical membrane of cholangiocytes and involved in the transport of organic anions such as bile acids. We were also able to detect the expression of alpha-acetylated tubulin in all cells, highlighting the presence of primary cilia, a sensory organelle present on the apical surface of polarized cholangiocytes, which plays an important role in modulating the secretory and proliferative functions of differentiated cells (Figure 3B).

**Figure 4:**
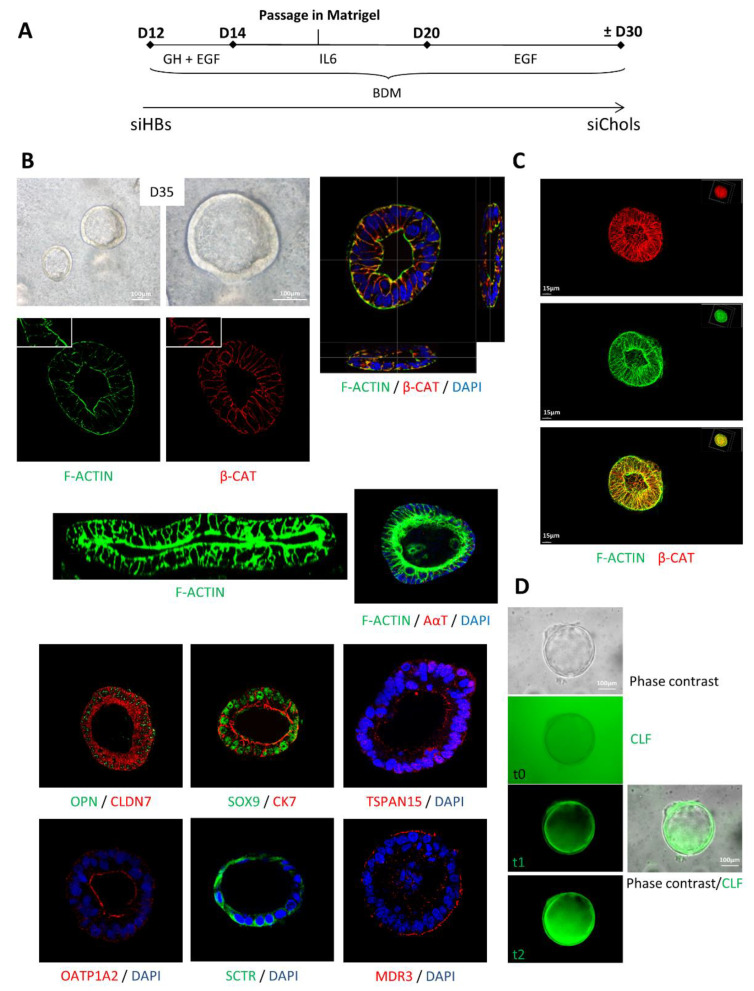
Formation of 3D siChol cysts. (**A**) Schematic representation of the protocol. (**B**) Cell morphology and immunostaining showing the expression of specific markers in cysts. Images were taken under the confocal microscope at 40X magnification. (**C**) 3D reconstruction of a siChol cyst using Imaris software. (**D**) Phase contrast and cholyl-lysyl-fluorescein (CLF) uptake showing progressive fluorescent bile acid analogue transport into the siChol cysts via organic anion transporting polypeptides (OATPs). BDM: biliary differentiation medium.

To further differentiate the cells, we generated 3D structures in hydrogel (Figure 4A). We observed round hollow structures composed of a layer of polarized cells (visualized by the beta-catenin labeling at the basolateral pole of the cells and F-actin at the apical side) (Figure 4B). In these structures, cells expressed cholangiocyte-specific markers such as OPN, SOX9, CK7, claudin-7 (a tight junction protein), OATP1A2, tetraspanin 15 and the secretin receptor. MDR3 (involved in bile acid efflux) was expressed on the basolateral membrane of the cholangiocytes. The 3D reconstruction confirmed the presence of a lumen within the cyst (Figure 4C). When the cysts were incubated with cholyl-lysyl-fluorescein—a fluorescent derivative of bile salts whose uptake into a cell is mediated by organic anion transporter proteins (OATPs) and apical secretion is mediated by multidrug resistance protein 2 (MRP2)—an accumulation of fluorescence was detected within the central lumen (Figure 4D).

## 3. Discussion

Our study reports on the generation of siPSCs from cynomolgus macaca somatic cells and their successful differentiation into liver cells. Using the same siPSCs, we generated hepatoblasts, hepatocytes and cholangiocytes, which has never been described before.

These cells express specific late markers of hepatocytes such as ALB and CK8 and display evidence of epithelial polarity, highlighted by ZO-1 and BSEP. The excretion of a fluorescent dye into bile canaliculi confirmed their functionality and Oil red and Periodic Acid-Schiff staining confirmed their capacity to store lipids and glycogen. The differentiation of siPSCs into iChols led to 3D structures of ciliated and polarized mature biliary cells expressing the specific markers SOX9, OPN, CK7, OATP1A2, CLDN7, TSPAN15, SCTR and MDR3.

Several studies showed that NHP livers are more similar to human livers than those of any other mammal [18], representing a good model to study liver development [19]. NHP models could also be very useful to assess the pharmacokinetics of drug candidates [20], which are difficult to study in mice or rats because of poor protein homology between their transporters and those of humans [21]. Another advantage of using NHPs for preclinical studies is the close similarity between the multiple forms of cytochrome P450 enzymes in monkeys and humans [22]. NHP liver disease models have also been developed for liver fibrosis [23] and acute liver failure [24], allowing the testing of new therapeutic approaches.

The use of liver cells in large animal models or humans requires a large quantity of cells and is therefore restricted by the difficulty of obtaining primary liver cells. The use of iPSC-derived cells represents a promising strategy for the massive generation of hepatocytes or cholangiocytes, but scaling up their production from established protocols requires optimization in several areas. Several strategies have been developed in order to upscale human iPSC and their derivative production and could be applied to simian iPSCs [25].

We have shown that siPSCs can be used to generate hepatoblasts, hepatocytes and cholangiocytes in vitro and that the differentiated cells can be genetically marked by GFP-expressing lentivectors for better in vivo post-transplant follow-up to study cell engraftment in mice and then in NHPs for preclinical studies (Supplementary Materials Figure S3). To date, all studies of liver cell transplantation in NHPs have used either human iPSC-derived liver cells or primary monkey liver cells [26]. Allografts in NHPs would permit the exploration of immune tolerance and requirements regarding immunomodulating regimens, essential questions in regenerative medicine.

However, before their use in preclinical studies, siChols and siHeps must be more extensively characterized in terms of functions and metabolism such as urea and bile acid production, cytochrome P450 activity, glucose metabolism and/or detoxifying abilities, and polarity.

Finally, siPSC-derived liver cells could be used for bioengineering approaches such as those developed with human cells [27]. Co-culture experiments using cells differentiated from the same siPSCs might even limit the immune response that would be caused by transplanting cell types from several different donors. All these features indicate that the use of iPSCs to generate liver cells is of considerable therapeutic interest.

## 4. Materials and Methods

### 4.1. Hepatoblast Differentiation of siPSCs

siPSCs were obtained as described [28], characterized using standard techniques (Supplementary Materials Figure S1) and seeded for differentiation as described in supplemental data. At day 0, the medium was replaced by RPMI/B27 supplemented with 50 ng/mL Activin A and 7.5 nM LY294002 (Stemcell Technologies, Vancouver, WA, Canada) for five days. The cells were then cultured with 25 ng/mL Activin A, 4 ng/mL FGF2 and 5 ng/mL BMP4 (R&D Systems, Minneapolis, MN, USA) for three days, and for four further days in methionine-deprived RPMI (Gibco, Invitrogen, Waltham, MA, USA) with 50 ng/mL HGF (Peprotech, Cranbury, NJ, USA) and 15 ng/mL FGF4 (Peprotech, Cranbury, NJ, USA).

### 4.2. Hepatocyte Differentiation of siHBs

siHBs were harvested using cell dissociation buffer (0.1 mg/mL EDTA, 0.5 mg/mL BSA) and seeded onto collagen I-coated plates in RPMI/B27, 10% Fetal Bovine Serum (FBS, Gibco, Waltham, MA, USA), 1% bovine serum albumin (BSA, Sigma, St. Louis, MO, USA), and 50 ng/mL HGF for four hours to enable cell attachment. The medium was then replaced by RPMI/ITS with 50 ng/mL HGF, 3 nM CHIR-99021 (Stemcell Technologies, Vancouver, WA, Canada) and 0.1 ng/mL TGF-β1 (Peprotech, Cranbury, NJ, USA), for 24 h. The medium was changed every day until the end of the differentiation with 20 ng/mL HGF, 0.05 nM dexamethasone (Sigma, St. Louis, MO, USA), 10 ng/mL oncostatin M (Peprotech, Cranbury, NJ, USA), 0.25 nM Compound E (Santa Cruz, Dallas, TX, USA), 2.5 nM SB431452 (Tocris, Bristol, UK) and 10 ng/mL vitamin K1 (Roche, Basel, Switzerland).

### 4.3. Cholangiocyte Differentiation of siHBs

The siHBs were harvested using StemPro™ Accutase™ (Gibco, Waltham, MA, USA) and seeded onto collagen I-coated plates in Williams’E (Invitrogen, Waltham, MA, USA), 10% FBS, 1% BSA. The medium was then replaced by a biliary differentiation medium (BDM) with 25 ng/mL Growth Hormone (GH, Peprotech, Cranbury, NJ, USA) and 25 ng/mL EGF, for two days. The medium was replaced daily by BDM with 5 ng/mL Interleukin-6 (IL6, Miltenyi Biotec, Bergisch Gladbach, Germany) for seven days and until the end of the differentiation with 10 ng/mL EGF. For 3D differentiation, siChols were harvested using Accutase, and 2 × 10^5^ cells were suspended in 100 µL 50:50 Matrigel (BD Biosciences, San Jose, CA, USA) and BDM on cell culture inserts with 1 µm pore size.

## Data Availability

Not applicable.

## Supplementary Materials

The supporting information can be downloaded at: https://www.mdpi.com/article/10.3390/ijms231810861/s1. References [1,29] are cited in the supplementary materials.
