# Supplementary material for: Successful Derivation of Hepatoblasts, Cholangiocytes and Hepatocytes from Simian Induced Pluripotent Stem Cells
Source: Int J Mol Sci. 2022 Sep 17;23(18):10861. doi: 10.3390/ijms231810861 (PMC9504404; doi:10.3390/ijms231810861)
Supplement: Supplementary file 1 [file ijms-23-10861-s001.zip › ijms-1854229-supplementary.pdf]

Supplemental Information

Supplemental Experimental Procedures

Preparation of culture medium

- RPMI/B27: RPMI-1640 medium (Gibco) with B27 supplement (Life technologies)(RPMI/B27), 1% non-essential amino acids (NEAA), 1% L-glutamine and 1% penicillin-streptomycin (all from Gibco).
- RPMI/ITS: 1% insulin-transferrin-selenium (ITS, Gibco), 0.1% BSA, 4.5mM nicotinamide, 0.1 nM zinc sulfate heptahydrate, and 10^{-5} M linoleic acid-Albumin (all from Sigma)
- BDM: Wiliams'E medium, 10mM nicotinamide, 17mM sodium bicarbonate, 0.2mM 2-Phospho-L-ascorbic acid trisodium salt, 14 mM glucose (all from Sigma), 6.3mM sodium pyruvate, 20 mM HEPES (all from Invitrogen), ITS, 1% L-glutamine and 1% penicillin-streptomycin.

Generation of simian iPSCs (siPSCs)

Fibroblasts from a three month old female cynomolgus monkey were obtained after skin punch biopsies and reprogrammed as previously described ***[1]***. Briefly, two rounds of retroviral transduction were performed using VSV-G pseudotyped retrovectors at low MOI (10^7 pfu/ml) in the presence of 8 µg/ml protamine sulfate (Sigma- Aldrich). The cells were treated after 24h with a combination of three small molecules (SPT: 2 mM SB431542, 0.5 mM PD0325901 and 0.5 mM thiazovinin). One day later, the cells were detached by treatment with trypsin and plated on irradiated mouse embryonic fibroblasts (MEFs) (GlobalStem), in α-MEM medium supplemented with 10% iFBS (Life Technologies). The medium was finally replaced, 24 h later, with standard human ESC medium containing 20% knockout serum (Gibco) and 4 ng/ml FGF2 (Peprotech).

Maintenance of undifferentiated simian iPSCs

Simian iPSCs were cultured in iPSC medium on irradiated MEF feeder cells (GlobalStem) at $3.5x10^5$ cells per 6 mm culture dish in a 5% CO2/5% O2 incubator. The cells were passaged every five days after 5 min of treatment with collagenase IV (0.1 mg/ml; Gibco).

Simian iPSC characterization

The siPSC line was characterized for stem cell marker expression and pluripotency by immunostaining and RT-PCR after *in vitro* differentiation. Karyotype integrity was confirmed.

Seeding of siPSCs for the differentiation

Three days before collection for seeding, siPSCs were treated with 100ng/ml Wnt3a (R&D). The cells were collected with trypsin-EDTA (0.5%) (Gibco) and incubated for 20 min on a gelatin-coated dish to remove the mouse embryonic fibroblast (MEF) feeder cells. The cells were then seeded onto 0.1% porcine gelatin pre-coated plates (Sigma) at 30,000 cells/cm² in MEF-conditioned medium with 10nM Y-27632 (Stemcell Technologies), 4ng/ml FGF2 (CellGenix) and 10ng/ml Activin A (Peprotech).

In vivo *pluripotency analyses of siPSCs*

The pluripotency of the siPSCs was assessed *in vivo* using teratoma assays. siPSCs cultured on MEFs were harvested manually by StemPro EZ passage (Invitrogen), collected by centrifugation and suspended in 120µl Geltrex (2mg/ml; Invitrogen) before injection into the thighs of NOD-SCID mice. Paraffin sections of the resulting teratomas were stained with hematoxylin and eosin. All animal procedures were approved by the local Ethics Committee.

Karyotype analysis

Karyotype analyses were carried out on cultured cells using standard procedures (RHG and GTG banding). Cells were cultured in StemMACS™ iPS-Brew XF supplemented with 0.02 mg/ml colchicine (Eurobio) for 2 h and then harvested and incubated with a warm hypotonic solution of 0.075 M KCl for 20 min. Finally, the siPSCs were fixed in Carnoy's fixative (methanol/acetic acid 3:1) and sent for analysis.

3D differentiation of siHBs

For 3D hepatocyte differentiation, 6-well plates were prepared as described elsewhere [29]. Briefly, sterilized powdered Ultrapure agarose (Life Technologies) was dissolved (2% w/v) by heating in sterile water. The liquid agarose solution was added to one well of a 6-well plate and left to solidify at room temperature while cylindrical micro-wells of 1 mm diameter and depth were formed using a home-made silicone mold. After cooling, the molds were lifted from the agarose gel and a single cell suspension of 2 x 10^6 cells/ml was added to the wells.

Immunostaining and fluorescent phalloidin staining

Immunostaining and fluorescent phalloidin staining experiments were performed on cell cultures in petri dishes and Matrigel and on whole spheroids. Fixation was performed with 4% paraformaldehyde for 20 min, permeabilization by incubation with 0.5% Triton (Sigma) in PBS for 15 minutes for 2D cultures, and HLC-spheroid sections and permeabilization solution (BSA 1% - 0.2µg/ml EDTA – 0.1% Triton) for spheroids. Under all conditions, blocking was performed with PBS-3%BSA for 20 min. The cells were then incubated overnight at 4°C with primary antibodies diluted in PBS-1%BSA (Table S1). Conjugated secondary antibodies were diluted to 1/1000, fluorescent phalloidin (Life Technologies – A12379) was diluted to 1/200 and all were incubated for 1 hour at room temperature in the dark. Cell nuclei were labeled with DAPI (0.5 µg/ml) (Sigma) and the samples were mounted in Faramount Aqueous mounting medium (Dako/Agilent Technologies). Images were obtained using the EVOS FL Auto microscope and its software (Thermo Fisher Scientific) or a confocal microscope Leica SP5 and its software for 3D imaging and treatment with ImageJ.

Flow cytometry

Flow cytometry analyses were performed after cell dissociation with trypsin-0.05% EDTA (Gibco), centrifugation and suspension in PBS-1%BSA. The cells were then incubated with conjugated antibodies for 30 minutes at 4°C (Table S1). Analyses were performed on a BD Accuri C6 Flow Cytometer (Becton Dickinson) and its software.

RNA extraction and RT-PCR

Total RNA was extracted using the RNAeasy mini kit (Qiagen) according to the manufacturer's protocol, and then reverse transcribed using the Superscript first-strand synthesis system (Invitrogen). PCR runs were performed with Platinum Taq DNA Polymerase (Invitrogen) (primer sequences in Table S2).

In vitro *assessment of differentiated hepatocyte functionality*

Oil Red O staining was assessed after the incubation of fixed cells with 60% isopropanol (Sigma) for 5 minutes followed by 60% Oil Red O (Sigma). The cells were then washed five times with distilled water and counterstained with hematoxylin (Sigma) for 2 minutes.

Periodic acid solution (PAS) staining was assessed after incubation of fixed cells with 1% PAS (Sigma) for 5 minutes. The cells were then incubated with Schiff's reagent (Sigma) for 15 minutes after four washes in distilled water, and counterstained with hematoxylin for 2 minutes.

In vitro *assessment of differentiated cholangiocyte functionality*

Bile acid transport into functional siPSC-derived cysts was visualized using 5µM cholyl-lysyl-fluorescein (CLF – BD Bioscience) for 15 min at 37°C. The cells were then washed twice with PBS and analyzed by microscopy using the EVOS FL Auto microscope and its software.

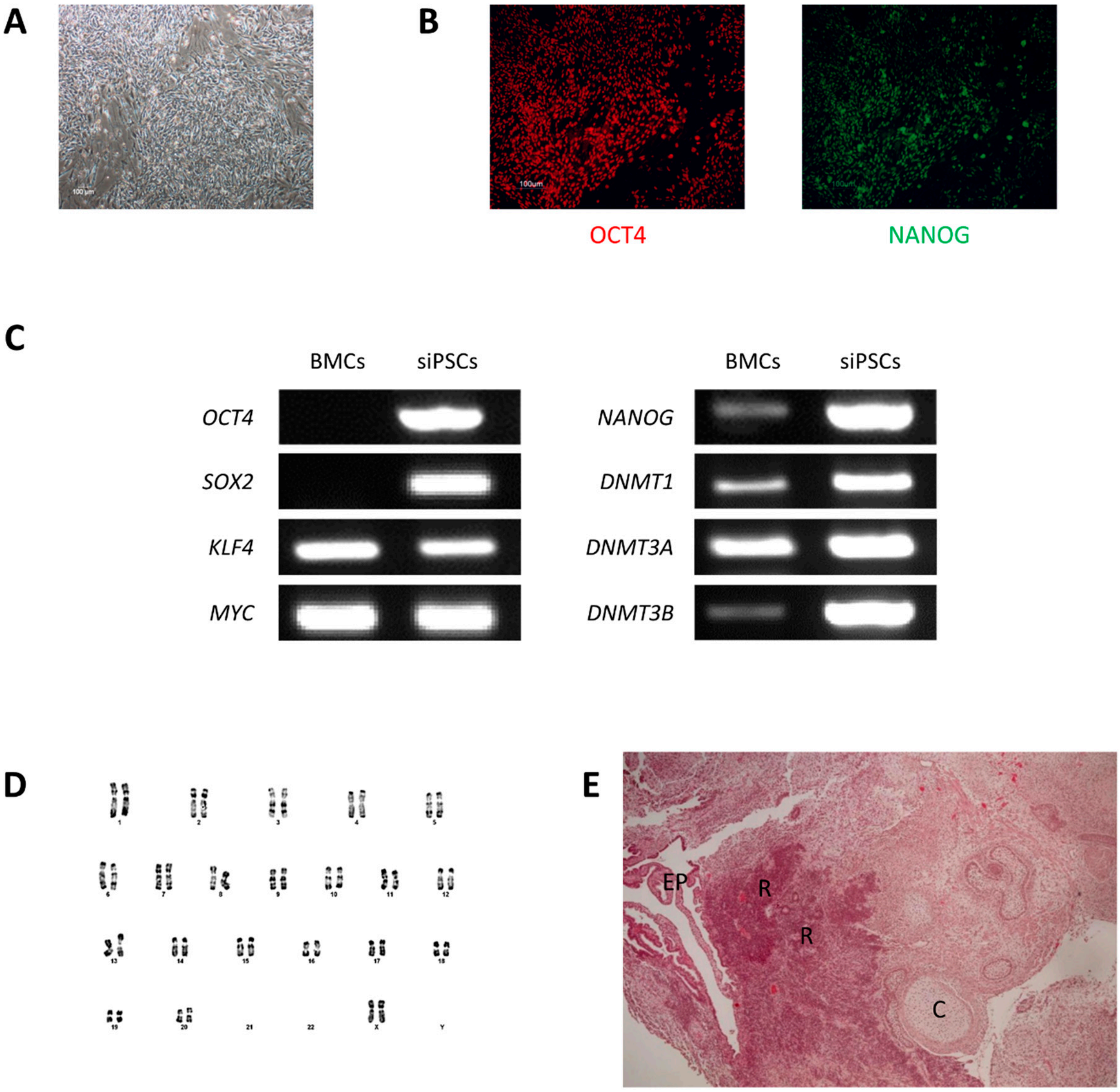

Supplementary Figure S1: Characterization of the simian iPSC line. (A) Morphology of siPSCs cultured on mouse embryonic fibroblast (MEF) feeder cells. (B) Expression of the stem cell markers OCT4 and NANOG by immunostaining in the siPSC line (x4 magnification). (C) RT-PCR showing the expression of several stemness-related genes in bone marrow-derived cells used for reprogramming and in the siPSCs (BMCs: bone marrow cells). (D) Normal karyotype: 40 X,X. (E) Histological analysis of teratomas obtained after the injection of siPSCs into immunodeficient mice and revealing the presence of derivatives of the three germ layers (EP: respiratory or intestinal epithelium derived from endoderm, R: rosette-like epithelium derived from ectoderm; C: cartilage derived from mesoderm).

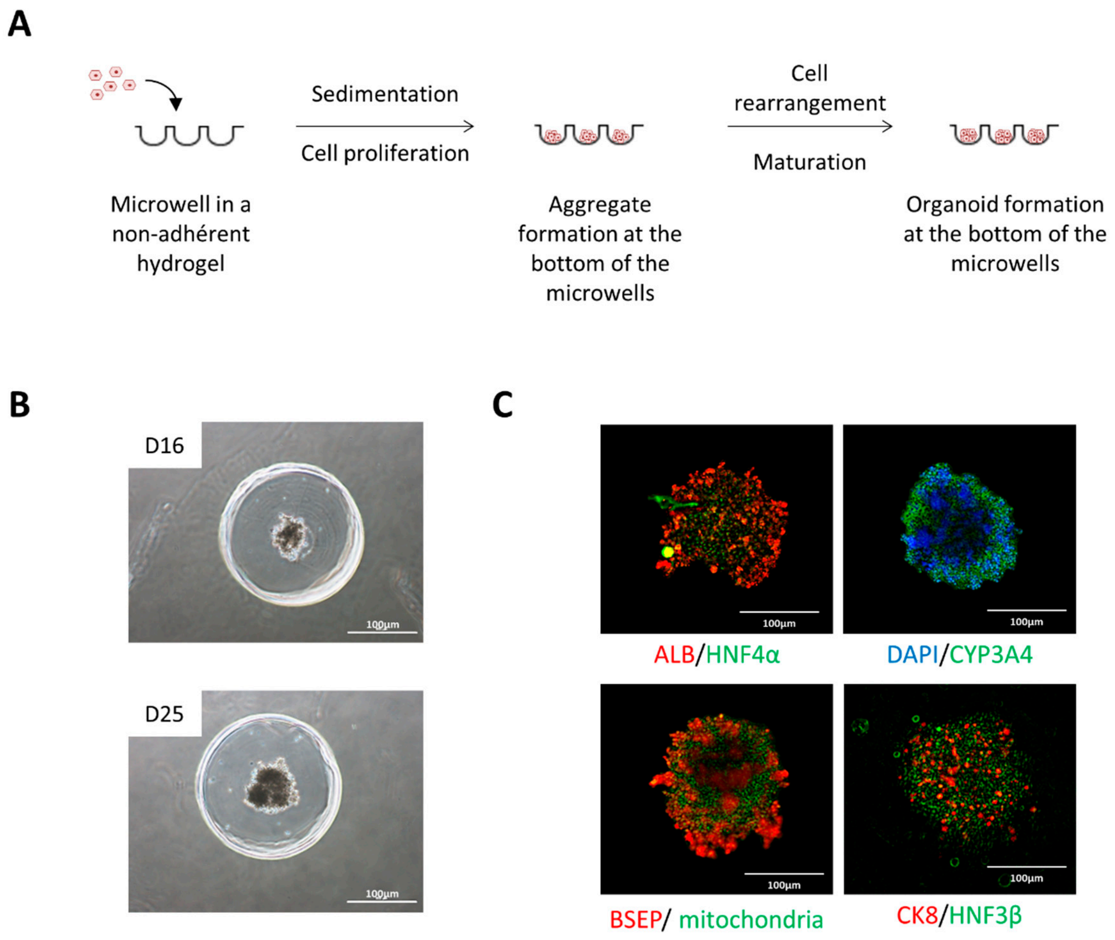

Supplementary Figure S2: siHep differentiation in aggregates. (A) Schematic representation of siHep aggregate formation. (B) Evolution of the siHep aggregate morphology from day 16 to day 25. (C) Immunostaining showing the expression of hepatocyte-specific markers in siHeps differentiated within aggregates. (ALB: albumin; BSEP: bile salt export pump; CK8: cytokeratin 8; CYP3A4: cytochrome P450 3A4; HNF4α and HNF3β: hepatocyte nuclear factor 4α and 3β).

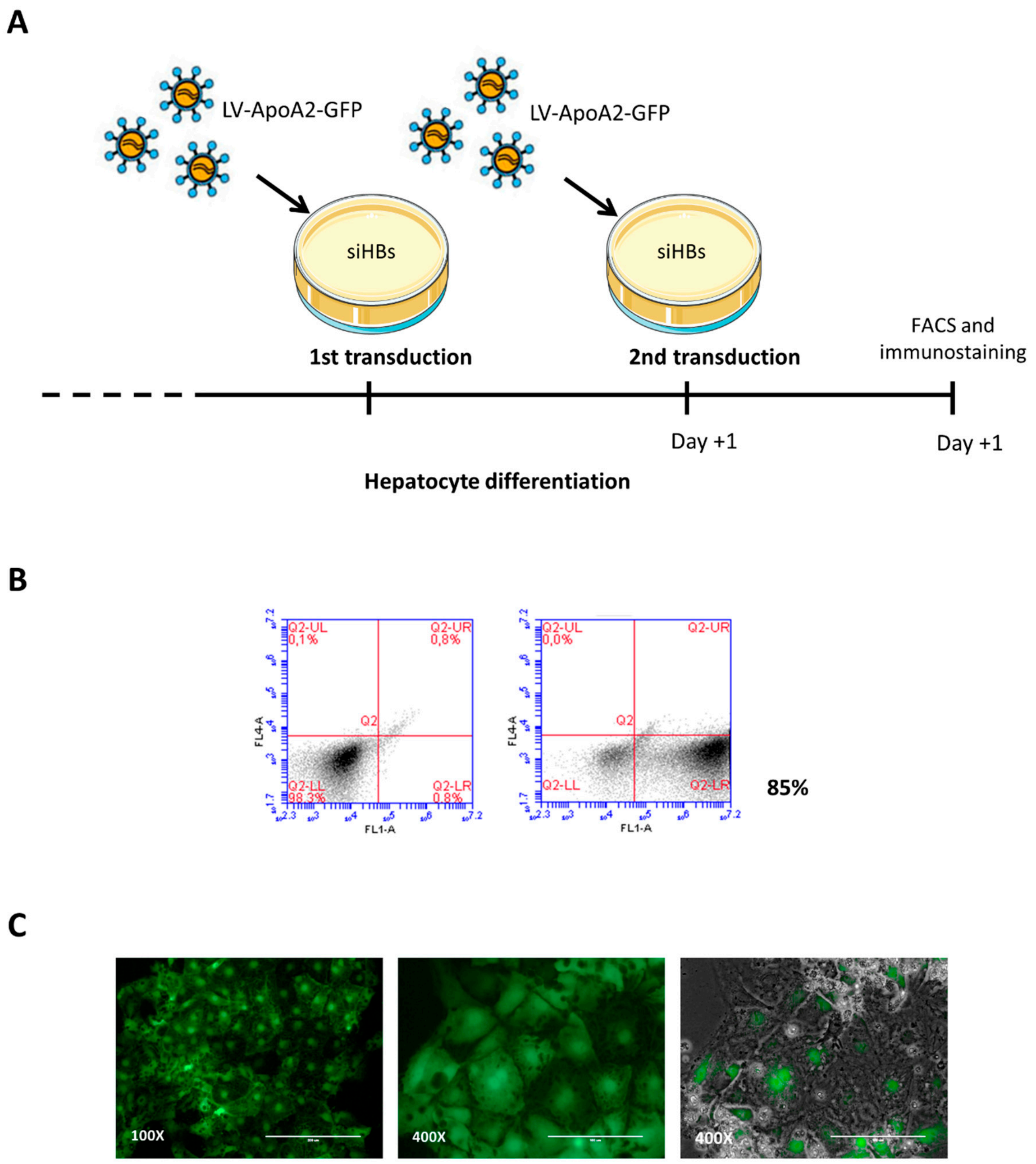

Supplementary Figure S3: GFP transduction of siPSC-derived hepatoblasts. (A) Schematic representation of the protocol. (B) Flow cytometry analysis showing 85% of GFP-positive siPSC-derived hepatoblasts. (C) Immunostaining showing the expression of GFP in transduced siPSC-derived hepatoblasts. (LV-ApoA2-GFP: lentiviral vector expressing GFP mRNA under the control of the ApoA2 liver-specific promoter).

Supplementary Table S1: List of primary antibodies used in this study.

Supplementary Table S2: List of RT-PCR primers used in this study.

Protein	Reference	Species	Dilution
**A1AT (α1 antitrypsin)**	DAKO - A0012	Rabbit	1/200
**AαT (acetylated α-tubulin)**	Sigma – T6793	Mouse	1/200
**AFP (α-fetoprotein)**	Santa Cruz - 8399	Mouse	1/200
**ALBUMIN**	Cedarlane - 2513A	Mouse	1/200
**ATP7B**	Novus Biologicals - 100-360	Rabbit	1/200
**β-CAT (β-CATENIN)**	Santa Cruz - sc-7963	Mouse	1/200
**BSEP (Bile salt export pump - ABCB11)**	Sigma - HPA019035	Rabbit	1/300
**CK7 (Cytokeratin 7)**	DAKO – M7018	Mouse	1/100
**CK8 (Cytokeratin 8)**	Santa Cruz - sc-8020	Mouse	1/200
**CK19 (Cytokeratin 19)**	DAKO - M0888	Mouse	1/200
**CLDN7 (Claudin-7)**	Abcam – ab27487	Rabbit	1/200
**CXCR4 (CD184)**	BD Pharmingen - 555976	Mouse	1/25
**CYP3A4**	Santa Cruz - 27639	Goat	1/200
**ECAD (E-CADHERIN)**	DAKO – M3612	Mouse	1/100
**GATA4**	Santa Cruz - sc-1237	Goat	1/200
**HNF3β**	Santa Cruz - sc-6554	Goat	1/200
**HNF4α**	Santa Cruz - 8987	Rabbit	1/200
**MDR3**	Chemicon - MAB4140	Mouse	1/200
**Mitochrondria**	Abcam - ab92824	Mouse	1/200
**NANOG**	R&D - AF1997	Goat	1/150
**OATP1A2**	Abcam – ab221804	Rabbit	1/200
**OCT4**	Santa Cruz – sc-5279	Mouse	1/200
**OPN (Osteopontin)**	Santa Cruz – sc-21742	Mouse	1/200
**SCTR (Secretin receptor)**	Santa Cruz - sc-166112	Mouse	1/200
**SREBP2 (Sterol regulatory element-binding protein 2)**	Abcam – ab28482	Rabbit	1/200
**SOX9**	Santa Cruz - sc-166505	Mouse	1/100
**TSPAN15 (Tetraspanin 15)**	Novus – NBP1-92540	Rabbit	1/200
**ZO-1**	Novus Biologicals - 85047	Rabbit	1/200

Table S1

	Simian Gene	Primer 5’ sequence	Primer 3’ sequence
RT-PCR	***ACTIN***	TTTTTGGCTTGACTCAGGATTT	GCAAGGGACTTCCTGTAACAAT
	AFP	TTTTGGGACCCGAACTTTCC	CTCCTGGTATCCTTTAGCAACTCT
	ALBUMIN	GTGAAACACAAGCCTAAGGCAACA	TCCTCGGCAAAGCAGGCCTC
	CD81	ATGACCCGCAGACCACCAACCT	TCCTTGGCGATCTGGTCCTTGT
	CK19	CCGCGACTACAGCCACTACT	GAGCTTGCTCGGTCTCAAAC
	DNMT1	GGGCTGGCCTCTATGGAAGGCTCGAG	AATGGCTTTGGCCAGGGGCGGCGGCAC
	DNMT3A	CGCTGGGTCATGTGGTTCGGAGA	CGCTGTCATGGCACACCGGGAACA
	DNMT3B	GGTGGAGGCAGACAGTGGA	TGGTACATGGCTTTTCGATAGG
	HNF3β	AAGTGGGGGTCGAGACTTTG	CTGCAACAACAGCAATGGAG
	HNF4α	CGGGTGTCCATACGCATCCTTG	GACCCTCCCAGCAGCATCTCCT
	KLF4	GATTACGCGGGCTGCGGCAAAAC	GTGTAAGGCGAGGTGGTCCGACC
	MYC	TGGTACTCCATGAGGAGACACC	CGCGTAGTTGTGCTGATGTGTGGAG
	NANOG	TCCAACATCCTGAACCTCAG	GACTGGATGTTCTGGGTCTG
	OCT4	GTGGAGGAAGCTGACAACAA	CAGGTTTTCTTTCCCTAGCT
	SOX2	AGCTACAGCATGATGCAGGA	GGTCATGGAGTTGTACTGCA

Table S2
